# Survival, Growth, and Food Resources of Juvenile Sea Cucumbers *Holothuria forskali* (Echinodermata, Holothuroidea) in Co-Culture with Shellfish in Brittany (France)

**DOI:** 10.1155/2024/7098440

**Published:** 2024-05-27

**Authors:** Frank David, Grégory Raymond, Julien Grys, Nadia Ameziane, Bastien Sadoul

**Affiliations:** ^1^ Direction Générale Déléguée à la Recherche l'Expertise la Valorisation et l'Enseignement (DGD REVE) Muséum National d'Histoire Naturelle (MNHN) Station Marine de Concarneau, Concarneau 29900, France; ^2^ Institut Agro Plateau Aquacole Expérimentation et Formation Station Marine de Concarneau, Concarneau 29900, France; ^3^ Institut de Systématique Évolution Biodiversité (UMR 7205 ISYEB) MNHN CNRS EPHE UA Station Marine de Concarneau Sorbonne Université, Concarneau 29900, France; ^4^ DECOD (Ecosystem Dynamics and Sustainability) Institut Agro Ifremer INRAE, Rennes, France

## Abstract

We conducted experiments with various growing conditions, both at sea and indoors, to explore the growth potential of *Holothuria* (*Panningothuria*) *forskali* Delle Chiaje, 1823 juveniles. Sea trials involved co-culture with European abalones (EA) or placement underneath European flat oysters (EO) or Pacific oysters (PO), using juveniles of 6–8 g initial weight. In sea-based conditions around summer (Apr–Sep), sea cucumbers grew best in EO at 0.94% d^−1^, followed by EA (0.88% d^−1^), both being in deep water (8–12 m), while sea cucumbers in the foreshore of a mega-tidal environment (PO) had the lowest growth (0.24% d^−1^). The indoor trial (IM) was performed with smaller individuals (≈0.3 g) and yielded a remarkable growth of 2.76% d^−1^ during summer (May–Sep). All experiments resulted in high survival rates, exceeding 80%. Additionally, we analysed fatty acid, amino acid, and stable isotope compositions of sea cucumbers' body walls, along with the pigment composition of their stomach contents. These analyses provided evidence that juveniles had distinct diets in each rearing condition, all differing from the diet of adults found in the wild. Our results also demonstrate that sea cucumbers do not compete for food resources in the shellfish production, which is crucial for their integration into multi-trophic aquaculture systems. However, whether sea cucumbers may have benefitted from the organic matter from shellfish faeces and pseudofaeces and/or grew on the biofilm growing on the cage walls remains to be elucidated.

## 1. Introduction

Global demand for seafood products has steadily increased over the last decades. Fishery captures have reached their maximum level in the 1990s with a nearly stable annual catch of 90 million metric tonnes [1]. To meet the rising demand, aquaculture production, almost negligible in the 1970s, began to grow in the 1980s and now accounts for approximately half of the total annual production of aquatic animals [1]. Yet, the strong and rapid growth of aquaculture is mainly driven by Asia, which now accounts for almost 90% of global production, in contrast to a mere 4% for Europe [1]. Multiple factors have been proposed over the last decades to explain the limited growth of aquaculture in Europe compared to Asia. The complexity of administrative procedures, the competition for space use, and the negative perceptions of aquaculture were among the most cited reasons [2, 3, 4]. Nevertheless, the production of shellfish has been historically well implanted, especially in France, the European leader in oysters' production, with nearly 81,000 metric tonnes produced in 2020 (82.5% of the EU production [5]). In pursuit of continuous improvement, researchers are now exploring the potential of integrated systems (integrated multi-trophic aquaculture—IMTA) to mitigate the environmental impacts of intensive productions, resolve conflicts related to coastal space usage, and enhance the social acceptance of aquaculture [6].

Sea cucumbers are considered excellent candidates for IMTA due to their assumed ability to feed on the waste of other organisms and their high market value [7, 8, 9, 10, 11, 12, 13]. Nevertheless, to date, most IMTA trials in Europe have involved adult sea cucumbers captured in the wild and have shown limited potential for bioremediation and growth from aquaculture waste [10, 14, 15, 16]. The induced breeding of European sea cucumbers and the early stages of their development was successfully achieved for a number of species, including *Holothuria arguinensis* [17], *Holothuria mammata* [18], *Holothuria tubulosa* [19], *Holothuria poli* [20], and *Holothuria forskali* [21]. Current trials are underway to investigate the growth potential of juveniles. Yet, wild populations are not well understood, and gaps remain regarding their food sources and growth rates [22, 23]. To address these gaps, we conducted opportunistic trials in collaboration with marine aquaculture producers to gain insight into the adaptability of juveniles of the sea cucumber *H. forskali* to various aquaculture conditions. The species has the particularity to live on rocky substrates, in contrast to most European sea cucumbers. Juveniles were stocked in addition or in replacement of the cultured shellfish without sediment or any material in the cages. Culture containers (cage/baskets) were those usually employed by the producers and commercially available. Targeted metabolomics were conducted to refine the food sources of juveniles and identify possible nutritional deficiencies. The objectives of the present study were to (i) evaluate the survival and growth of juvenile sea cucumbers in co-culture with shellfish at sea, (ii) compare food sources of juveniles from different growing conditions, and (iii) compare the nutritional composition of reared individuals and identify strengths and weaknesses of each growing system.

## 2. Materials and Methods

### 2.1. Juveniles Production

**Figure 1:**
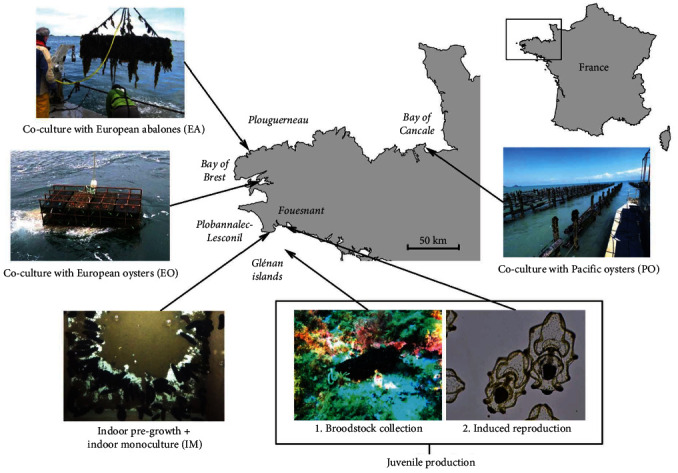
Localisation of the various steps of *H. forskali* rearing during the HoloFarm research project in Brittany (France).

Adult sea cucumbers of the species *Holothuria* (*Panningothuria*) *forskali* Delle Chiaje, 1823 were sampled on various occasions in early spring 2019 east of the Glenan Islands (Brittany—France; 47.710°N, 3.948°W; Figure 1) at 8–12 m deep and brought to the marine experimental station of “L'Institut Agro” in Fouesnant (Brittany) for spawning induction. We induced spawning by thermal stimulation on the 30th of April 2019, following the method previously described by our team [21]. In short, the water temperature was quickly raised from 15–16°C to 18–19°C by a continuous flow of 1 *μ*m filtered seawater and was maintained at 18–19°C until spawning. When spawning occurred, fertilised eggs were removed from the tank with a polyamide sieve (80 *μ*m mesh size) and washed with seawater. The seawater used for embryonic and larval rearing was sand filtered, then filtered with a 1 *μ*m filter bag and sterilised by UV light. Eggs were incubated at 18–18.5°C for 12 hr in aerating 10 L flat bottom trays at a density of approximately 12,500 embryos L^−1^. Larvae were transferred to aerating 35 L cylindroconical tanks and maintained, at a density of 5–10 larvae mL^−1^, in closed-water systems at 17–18.5°C with natural photoperiod. Seawater of hatchery tanks was renewed every 2 days with partial seawater change (50%) using an 80 *μ*m mesh sieve. Between the late gastrula and the early auricularia stage, larvae were fed daily with a mixture of the microalgae *Isochrysis galbana* and *Chaetoceros calcitrans* (1 : 1 in cell numbers) at a final concentration of 1–4.10^4^ cell mL^−1^. From the late auricularia stage until the doliolaria stage, they were daily fed with *I. galbana*, *C. calcitrans*, and *Phaeodactylum tricornutum* (1 : 1 : 1 in cell numbers) at the same final concentration. Microalgae were cultivated in Conway growth medium, successively in 500 mL, 2 L, 10 L Erlenmeyers, and 310 L cylinder, using the method of the batch culture of microalgae. Larvae were moved to aerating 24 L flat bottom trays once the first pentacula larvae appeared in the cylindroconical tanks. From then on they were fed with scratched Plexiglas plates colonised with the benthic diatoms *Nitzschia alexandrina* and *Staurosira elliptica* until being transferred to the pre-growth phase. The survival rate between the oocyte and juvenile stage was around 11% (Raymond and Grys, unpublished data).

### 2.2. Experimental Designs

#### 2.2.1. Indoor Pre-Growth

Young individuals (≈5 mg) were transferred from “L'Institut Agro” to “Aqua B” company in Plobannalec-Lesconil (Brittany) for a pre-growth phase on the 16th of January 2020 (Figure 1). Juveniles were simply transported in aerating 30 L plastic tubs without any observed adverse effects. The “Aqua B” company maintained the juveniles in 2.5 m long and 0.5 m wide raceway tanks filled with 50 cm of heated seawater (20 ± 1°C) at a density of 80 individuals m^−2^ and fed them with decomposed green macroalgae (*Ulva* sp. or *Enteromorpha* sp.) every 2 to 3 days ensuring *ad libitum* conditions (food always available in significant amounts). Green seaweeds were gathered on nearby beaches and left to decompose during 1 month in aerated cylindroconical tanks, and the mixture was filtered to select particles between 50 and 300 *μ*m for feeding. Algae were spread as the water surface at the end of the feeding days, and water circulation was stopped overnight to ensure proper deposition in the tanks (Figure 1). Unconsumed food and faeces were left in the raceways as previous observations have shown that *H. forskali* juveniles could feed and grow on their own faeces (Grys and Raymond, unpublished data). Tanks were cleaned once a month to prevent excessive microbial development and anoxic areas, and 10% of water was renewed weekly.

#### 2.2.2. Co-Cultured with European Abalones (EA Condition)

A first batch of pre-grown sea cucumbers (*n* = 80; weight = 6.2 ± 1.0 g) were transferred from “Aqua B” company to “France Haliotis” company in Plouguerneau (Brittany) on the 25th of November 2020 for a 9 months co-culture experiment with EA, *Haliotis tuberculata* (Figure 1). Juveniles were individually weighed and placed in two 1 m^3^ cubic polyethylene cages with 6 mm wide vents (40 sea cucumbers and 600 abalones weighing 15–20 g per cage), deposited at the bottom of the Aber Wrac'h estuary (8–12 m deep—Brittany) and anchored on floating long lines. Cages were pulled up once a month, and 10–15 kg of fresh red and green macroalgae (*Palmaria palmata*, *Saccharina latissima*, and *Laminaria digitata*) were added. This followed the classical rearing protocol of “France Haliotis” for growing abalones in monoculture. An intermediate biometry was performed on a subsample of 28 *H. forskali* randomly sampled in one cage on the 7th of April 2021 (i.e., after 4 months). At the end of the experiment (2nd of September 2021, 9 months of co-culture), all sea cucumbers were weighed individually, and seven random individuals were transported in water to the laboratory and frozen within 6 hr of collection. Dissections were performed on thawed individuals to sample body wall and stomach contents. Individuals were dorsally incised, and the entire stomach was carefully removed and emptied with plastic vials. The remaining viscera were discarded, muscular bands were torn off, and the central third of the body wall was kept. Samples were then rapidly stored at −25°C for further freeze–drying and analyses.

#### 2.2.3. Co-Cultured underneath European Flat Oysters (EO Condition)

A second batch of pregrown sea cucumbers (*n* = 18; weight = 6.7 ± 1.2 g) was transferred from “Aqua B” company to “Keraliou” company in Plougastel–Daoulas (Brittany) on the 22nd of March 2021 for a 6 months co-culture experiment below EO, *Ostrea edulis* (Figure 1). The 18 juveniles were individually weighed and placed in three commercial 85 × 50 × 10 cm^3^ and 6 mm mesh size rigid plastic baskets (six individuals per basket with no oysters), arranged 20 cm underneath four layers of identical baskets containing 3.5 kg of flat oysters each and 40 cm above the sediment using a metal structure. The entire structure was anchored at 8–12 m deep in the Bay of Brest. At the end of the experiment (16th of September 2021, 6 months of co-culture), sea cucumbers were weighed individually, and six individuals were transported in water to the laboratory and frozen within 24 hr of collection. Dissections were performed on thawed individuals to sample body wall and stomach contents, and samples were rapidly stored at −25°C for further freeze–drying and analyses. Given that individuals were frozen a relatively long time after collection, only three individuals presented contents in their stomachs.

#### 2.2.4. Co-Cultured underneath Pacific Oysters (PO Condition)

A third batch of pregrown sea cucumbers (*n* = 81; weight = 7.5 ± 1.3 g) was transferred from “Aqua B” company to “Les Parcs Saint Kerber” company in Cancale (Brittany) on the 29th of April 2021 for a 4 months co-culture experiment below PO, *Magallana gigas* (Figure 1). The 81 juveniles were individually weighed and placed in nine commercial plastic baskets of 15 L volume (15 × 20 × 60 cm^3^) with hexagonal prism shape and 6 mm rigid mesh. These baskets are commonly called Australian oyster baskets. The baskets were hanged using lines stretched between sediment-pounded poles on the foreshore of the Bay of Cancale and 120 cm underneath three layers of identical baskets, each containing 6 kg of oysters. The baskets containing sea cucumbers were positioned sufficiently deep on the foreshore to prevent them from emerging throughout the experiment. They were also placed 10 lines inside (40 m) from the edges of the oyster field to ensure that they received inputs from oyster dejections from all directions. It's worth mentioning that the farm is located in one of the regions of the world with the highest tidal range, with water height variations of up to 14 m during spring tides. At the end of the experiment (9th of September 2021, 4 months of co-culture), sea cucumbers were weighed individually, and seven individuals were transported in water to the laboratory and frozen within 6 hr of collection. Dissections were performed on thawed individuals to sample body wall and stomach contents, and samples were rapidly stored at −25°C for further freeze–drying, and analyses.

#### 2.2.5. Indoor Monoculture (IM Condition)

Another batch of young individuals (*n* = 240; weight = 0.33 ± 0.04 g) from different spawns performed in spring 2020 (i.e., 1 year later compared to other batches) was transferred from “L'Institut Agro” to “Aqua B” company in Plobannalec–Lesconil for a 4 months monoculture experiment on the 4th of May 2021 (Figure 1). The 240 juveniles were weighed in batches and placed in three 2.5 m long, 0.5 m wide, and 50 cm deep raceway tanks. Water parameters and feeding conditions were similar to those of the pre-growth phase. At the end of the experiment (6th of September 2021, 4 months of monoculture), sea cucumbers were weighed individually, and eight individuals were immediately dissected. Body walls, stomach contents, and feed samples were isolated and placed at −25°C for further freeze–drying, and analyses.

#### 2.2.6. Wild Collection

Wild *H. forskali* (*n* = 6; weight = 199.6 ± 41.8 g) were collected on the 28th of September 2020 at the same site as the broodstock sampling site (east of the Glenan Islands). The biochemical composition of these individuals' body wall (kept at −25°C until freeze–drying and analyses) was compared with reared juveniles.

### 2.3. Biometric and Biochemical Analyses

#### 2.3.1. Weight Measurements

Biometric measurements of sea cucumbers are subject to debate as the plasticity of their body makes precise measures difficult, and the presence of water in their body cavity may alter wet weight measurements by more than 30% [24]. We aimed at reducing the stress of individuals as much as possible during handling to avoid cuverian tubules excretion and further energy losses due to their regeneration. Juveniles were thus weighed by rapidly transferring them from their transport container to a pre-weighed 1 L plastic jar filled with transport seawater. Juveniles below 1 g were weighed in batches of 30–80 individuals, and those above 1 g were weighed individually.

#### 2.3.2. Pigments Determination

Pigments were analysed by high-performance liquid chromatography (HPLC) according to Brotas and Plante-Cuny [25]. We disrupted 5–15 mg of freeze–dried stomach contents or feed samples (IM) in 1.8 mL of 95% methanol (buffered with 2% w : v ammonium acetate) with a stainless steel bead in a 2 mL plastic tube using a MM 400 Retsch bead mill at 30 Hz for 10 min. Extracts were then filtered with 0.2 *μ*m PTFE syringe filters and analysed within 16 hr using an Agilent 1260 Infinity HPLC composed of a quaternary pump (VL 400 bar), a UV–VIS photodiode array detector (DAD 1260 VL, 250–900 nm) and a 100 *μ*L automatic sample injector refrigerated at 4°C in the dark.

Chromatographic separation was carried out using a C18 column for reverse phase chromatography (Supelcosil, 25 cm long, 4.6 mm inner diameter). The solvents used were A: 0.5 M ammonium acetate in methanol and water (85 : 15, v : v), B: acetonitrile and water (90 : 10, v : v), and C: 100% ethyl acetate. The solvent gradient was set according to Brotas and Plante-Cuny [25] with a 0.5 mL min^−1^ flow rate. Identification and calibration of the HPLC peaks were performed with *β*-caroten, canthaxanthin, chlorophyll *a*, chlorophyll *b*, diatoxanthin, diadinoxanthin, fucoxanthin, and pheophytin *a* standards. We identified all detected peaks by their absorption spectra and relative retention times using the Agilent OpenLab software. Quantification was performed using standard calibration curves built with repeated injections of standards over a range of dilutions. Carotenoids and chlorophyll *b* were quantified at 470 nm, chlorophyll *a* and their derivatives, as well as pheopigments were quantified at 665 nm. The relative abundance of each pigment (%) was calculated from its respective concentration in the sample (mg g^−1^ of dry weight sample).

#### 2.3.3. Amino Acid (AA) Quantitation

Due to its hard and elastic nature, the body wall could not be crushed finely with mortar and pestle. Instead, samples of freeze-dried body wall for AA, fatty acid (FA), and isotopic analyses were cut in fine transverse slices (max 1 mm thickness) with a sharp knife. AAs were quantified by HPLC using 8–12 mg of body wall. Samples were placed in glass ampules, and 200 *μ*L of HCl (6 N) was added. The acid mixture was carefully degassed with liquid nitrogen to reduce the level of oxidative destruction. Ampules were sealed, and proteins were hydrolysed in vacuum at 110°C during 24 hr, opened, and dried with a speedvac. Free AAs were reconditioned in Pickering diluent prior to injection in the HPLC system. AAs were separated by ion exchange HPLC using a high-efficiency sodium column (4 × 150 mm; Pickering Lab, LCTech, Dorfen, Germany) with a Waters 2695 separation module (Waters). The elution buffers and gradient conditions were those recommended by the manufacturer (detailed in [26]).

Free AAs were first subjected to postcolumn derivatisation with ninhydrin (Pickering Lab.) by using a PCX 5200 derivatiser (Pickering Lab.) and later detected on a Waters 2996 photodiode with a UV module detector at 570 nm for all the AAs containing a primary amine, and at 440 nm for the proline which holds a secondary amine. Quantification was performed by repeated injections of standards over a range of dilutions to determine the relationship between peak area and standard concentrations in mg AA per g of the freeze-dried body wall. Protein concentration was calculated as the sum of AA concentrations. Note that tryptophan was degraded during hydrolysis and thus not considered, while asparagine and glutamine were respectively converted to aspartic acid and glutamic acid during hydrolysis.

#### 2.3.4. FA Profiling

We analysed FAs following a slightly modified method of Bligh and Dyer [27] as developed by Meziane et al. [28] and adapted by David et al. [22]. Lipids were extracted from 50–75 mg of body wall or 20–40 mg of feed using a methanol : water : chloroform mixture (2 : 1 : 2 v :v : v) after adding 30 *μ*g of tricosanoic acid (23 : 0) provided by Sigma–Aldrich as internal standard. Chloroform, containing lipid extracts, was isolated, and evaporated under nitrogen. Dried lipid extracts were then saponified with a solution of methanol and sodium hydroxide (NaOH, 2 N) (2 : 1, v : v) at 90°C for 1 hr 30 min. Subsequently, methylation was performed using methanolic boron trifluoride 14% (BF_3_—CH_3_OH).

FA methyl esters (FAME) were analysed by gas chromatography (Agilent, 7890B GC) equipped with an Agilent VF-WAXms capillary column (30 m length × 0.25 mm inner diameter × 0.25 *μ*m film thickness) and quantified using a flame ionisation detector (FID). The oven temperature was set at 60°C and held for 1 min, raised at 40°C min^−1^ to 150°C and held for 3 min, and then increased to 240°C at 3°C min^−1^ and held for 8 min. We identified FAs using coupled gas chromatography with simple quadrupole mass spectrometry (Agilent, 5977B MSD) using the NIST mass spectral library and comparison of GC retention times with commercial standards (Supelco® 37 component FAME mix). The internal standard (23 : 0) was used to determine the concentration of each FA in mg FA per g of freeze–dried body wall.

#### 2.3.5. Stable Isotope Analysis

We performed isotopic analyses in tin capsules using 0.2–1 mg of body wall. Thin vertical sections of the body wall were divided in two. Only the inner part of these sections was used for isotopic analysis, as previous observations revealed that calcareous ossicles were restricted to the outer part of the body wall (Badou et al., in preparation). We also analysed stomach contents and feed samples of the IM trial to calculate the trophic enrichment factor of this species. Stable isotopes were analysed at the Institut Universitaire Européen de la Mer in Brest (Brittany) using a Flash EA 2000 IRMS elemental analyser interfaced to a Delta V Plus isotope ratio mass spectrometer. Carbon and nitrogen stable isotope ratios were reported in delta notation for *δ*^13^C and *δ*^15^N relative to Vienna PeeDee Belemnite and atmospheric air, respectively.

### 2.4. Statistical Analysis

We calculated specific growth rates (SGR) as the percentage increase in sea cucumber weight per day using the formula SGR (% d^−1^) = 100 × ln (*W*_*t*_/*W*_0_) /*t*, where *W*_*t*_ is the average weight of all individuals at the end of the period, *W*_0_ is the average weight of all individuals at the beginning of the period, and *t* is the number of days of the period. We performed multiple comparisons of total concentrations (pigments, AAs, FAs) using the non-parametric Van der Waerden test (R package “agricolae”) due to small sample sizes (*n* = 3–8). We visualised the underlying structure of biochemical compound assemblages using principal component analysis (PCA) on standardised datasets. Pigments database consisted of absolute concentrations in stomach contents, while compounds measured in the body wall (AAs and FAs) were treated as relative proportions. Statistical analysis and graphical representations were performed using R 4.1.2 (R Core Team 2021), and type I error (*α*) was set to 5%.

## 3. Results

### 3.1. Survival and Growth

All experiments resulted in high survival rates (>80%; Table 1) and efficient growth. Sea cucumbers reared with EA showed 93% survival despite the long experimental duration, and multiplied their body mass by 5.3 (Table 1). The specific growth rate was substantially higher in spring and summer (EA condition; SGR = 0.88% d^−1^; final weight = 33.2 ± 7.1 g) compared to winter (SGR = 0.28% d^−1^; intermediate weight = 9.1 ± 2.8 g). Underneath oysters, sea cucumbers showed nearly no mortality (99%–100% survival), but growth was lower in the foreshore below PO condition (SGR = 0.24% d^−1^; final weight = 10.3 ± 3.1 g) compared to 8–12 m deep below European oysters (EO condition; SGR = 0.94% d^−1^; final weight = 35.3 ± 6.6 g). Finally, indoor rearing (IM) showed lower survival (84%, Table 1), but sea cucumbers were also smaller and, therefore, more at risk (early live stages are generally subject to higher mortality in cultivated species). Assuming that sea cucumbers follow a von Bertalanffy growth curve, smaller initial size also contributed to higher growth rates (IM condition; SGR = 2.76% d^−1^; final weight = 10.3 ± 6.3 g), with juveniles reaching a size similar than sea cucumbers co-cultured with PO, multiplying their body mass by 31.5.

### 3.2. Pigments of Stomach Contents

**Figure 2:**
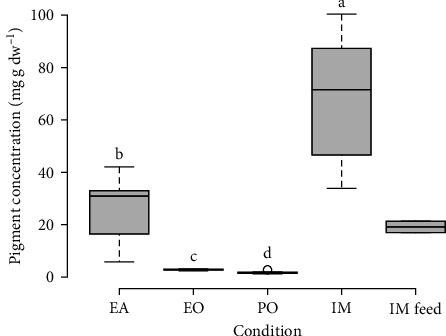
Total pigment concentrations in stomach contents of *H. forskali* juveniles co-cultured at sea and in indoor monoculture (IM) and its feed. EA, EO, and PO stand, respectively, for co-culture with European abalones, European oysters, and Pacific oysters. Letters indicate significant differences between samples (Van der Waerden test, *α* = 5%). Broad lines indicate the median, box edges refer to the 1st and 3rd quartiles, and circles indicate outliers.

The HPLC system identified 25 different pigments in stomach content and feed samples. Yet, most compounds could not be precisely identified as the corresponding purified standard was not available. Most minor pigments were derivatives of major ones, and thus, they were combined with their closest relative compound. One exception was fucoxanthin, whose derivatives did not seem to co-vary with the pure compound. Derivatives were thus combined in a separate group. The highest pigment concentration in stomach contents was measured in the IM condition (68.2 ± 24.6 mg g dw^−1^; Figure 2). Concentration was more than three times higher in stomach contents compared to feed in the IM condition (19.2 ± 3.1 mg g dw^−1^). Every condition significantly differed from one another (Van der Waerden test; *p*  < 0.001; Figure 2). The EA condition exhibited intermediate concentrations (25.4 ± 12.9 mg g dw^−1^) while EO and PO conditions exhibited the lowest values (respectively 2.8 ± 0.4 and 1.8 ± 0.5 mg g dw^−1^).

**Figure 3:**
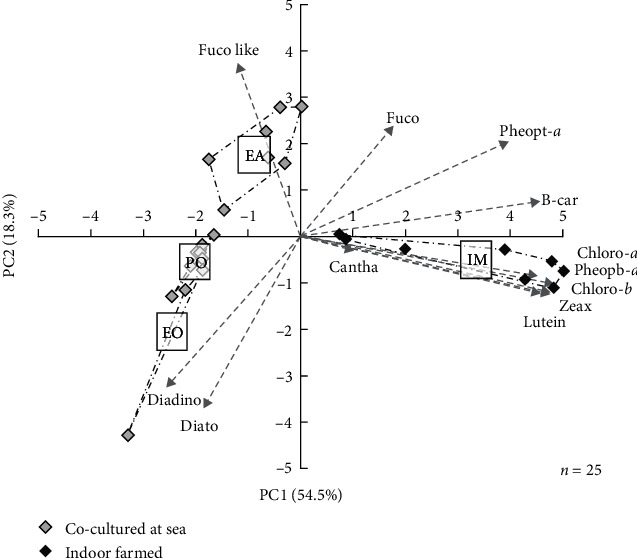
Principal component analysis of pigment concentrations in stomach contents of *H. forskali* juveniles co-cultured (grey squares) underneath European oysters (EO) or Pacific oysters (PO), with European abalone (EA) or indoor farmed (IM, black squares). Pigments most contributing to the construction of axes are represented by vectors.

The PCA analysis clearly discriminated rearing conditions based on the pigment profile of sea cucumber stomach contents (Figure 3). The IM condition was characterised by high levels of chlorophyll *a* and *b*, lutein, zeaxanthin, and degradation products of chlorophyll *a* (pheophytin *a* and pheophorbide *a*) (Table 2). These compounds are typical biomarkers of green algae. In the EA condition, prominent biomarkers were fucoxanthin derivatives, which are usually particularly abundant in brown algae. Stomach contents of both EO and PO conditions exhibited similar pigment composition, especially rich in diatoxanthin and diadinoxanthin, which are commonly found in diatoms.

### 3.3. Biochemical Compounds

#### 3.3.1. AAs

**Figure 4:**
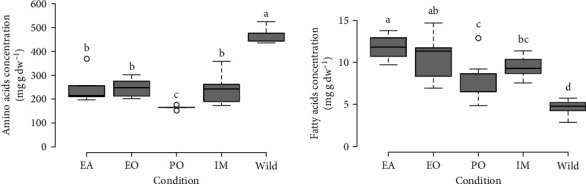
Total amino acid and fatty acid concentrations in the body wall of *H. forskali* juveniles co-cultured at sea, in indoor monoculture, or in adults gathered in the wild. EA, EO, and PO stand, respectively, for co-culture with European abalones, European oysters, and Pacific oysters. Letters indicate significant differences between samples (Van der Waerden test, *α* = 5%). Broad lines indicate the median, box edges refer to the 1st and 3rd quartiles, and circles indicate outliers.

The HPLC system identified 16 different AAs in body wall samples of *H. forskali*. Glycine, glutamic acid, alanine, and proline were the dominant AA. The highest total AA concentration (i.e., protein percentage) in the body wall of *H. forskali* was measured for wild individuals (465.0 ± 37.3 mg g dw^−1^; Figure 4). Reared juveniles exhibited average total AA concentration ranging from 165.0 ± 8.5 mg g dw^−1^ (PO condition) to 249.4 ± 70.5 mg g dw^−1^ (EA condition). Significant differences were revealed between wild sea cucumbers and reared juveniles and between individuals co-cultured underneath PO and other conditions (Van der Waerden test; *p*  < 0.001; Figure 4).

**Figure 5:**
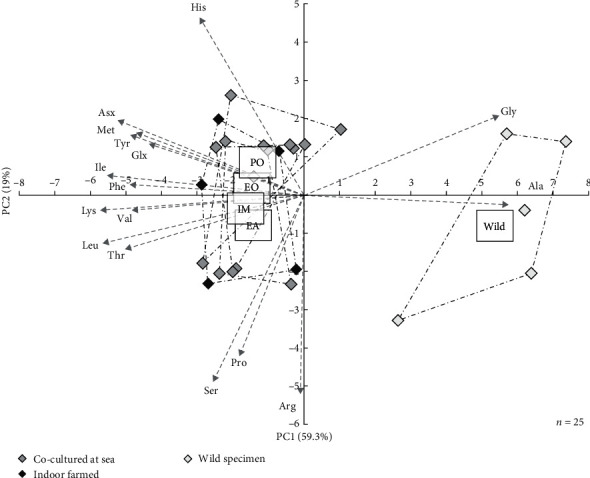
Principal component analysis of amino acid proportions in the body wall of *H. forskali* juveniles co-cultured (grey squares) underneath European oysters (EO) or Pacific oysters (PO), with European abalone (EA) or indoor farmed (IM, black squares). Values were compared to adults collected in the wild (white squares). Amino acids, most contributing to the construction of axes, are represented by vectors.

The PCA analysis clearly discriminated wild sea cucumbers from reared juveniles according to the AA profile of their body wall (Figure 5). Adult sea cucumbers from the wild exhibited higher relative proportions of glycine, a major component of collagen, and alanine, and consequently lower relative proportions of all other 14 detected AA (Table 3). Yet, rearing conditions could not be differentiated according to the AA profile of sea cucumbers body wall.

#### 3.3.2. FAs

The GC-FID/MSD system identified 29 different FAs in body wall samples of *H. forskali*. Major FA were 20 : 5*ω*3, 20 : 4*ω*6, and 20 : 1*ω*9 (Table 4). The lowest total FA concentration was measured in wild individuals (4.6 ± 1.0 mg g dw^−1^; Figure 4). Reared juveniles exhibited average total FA concentration ranging from 7.8 ± 2.6 mg g dw^−1^ (PO condition) to 11.8 ± 1.5 mg g dw^−1^ (EA condition). Significant differences were revealed between wild sea cucumbers and reared juveniles and between farming conditions (Van der Waerden test; *p*  < 0.001; Figure 4).

**Figure 6:**
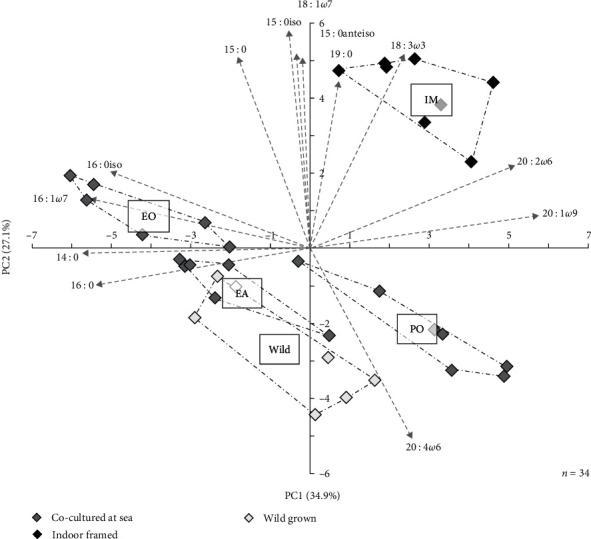
Principal component analysis of fatty acid proportions in the body wall of *H. forskali* juveniles co-cultured (grey squares) underneath European (EO) or Pacific oysters (PO), with European abalone (EA) or indoor farmed (IM, black squares). Values were compared to adults collected in the wild (white squares). Fatty acids most contributing to the construction of axes are represented by vectors.

The PCA analysis clearly discriminated juvenile sea cucumbers reared indoors (IM condition) from those grown at sea (ranched or wild) according to the FA profile of their body wall (Figure 6). Juveniles of the IM condition were characterised by higher relative proportions of the FA 18 : 3*ω*3, which is a common biomarker of green algae, and branched, odd-chain FA (15 : 0) and the FA 18 : 1*ω*7, which are bacterial biomarkers and especially abundant in the feed (Table 4). Juveniles co-cultured underneath European oysters (EO condition) exhibited especially high levels of the FA 16 : 1*ω*7, commonly assumed to derive from diatoms, while those co-cultured underneath PO condition were characterised by higher proportions of highly unsaturated FA (HUFA; ≥20 carbons chain length and three double bounds).

#### 3.3.3. Stable Isotopes

**Figure 7:**
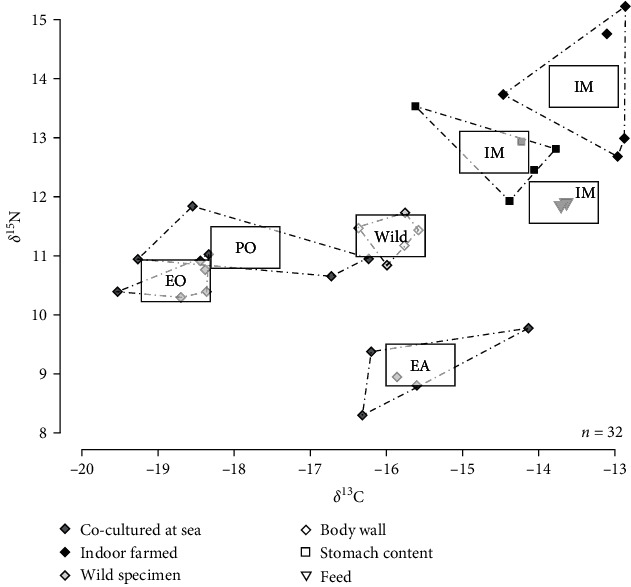
Stable isotope ratios (*δ*^13^C and *δ*^15^N) in the body wall and in stomach contents of *H. forskali* juveniles co-cultured (grey squares) underneath European (EO) or Pacific oysters (PO), with European abalone (EA) or indoor farmed (IM, black squares). Values were compared to adults collected in the wild (white squares) and the feed (triangles) provided to indoor-farmed individuals.

The lowest *δ*^13^C values were measured in the body wall of juvenile sea cucumbers co-cultured underneath European oysters (EO condition; −18.7 ± 0.5‰; Figure 7), while most enriched values were obtained in IM condition (−13.3 ± 0.7‰). The lowest *δ*^15^N values were measured in the body wall of juvenile sea cucumbers co-cultured with the EA condition (9.0 ± 0.6‰), while most enriched values were obtained in the IM condition (13.9 ± 1.1‰). Stomach contents of sea cucumbers grown in IM condition had slightly lower *δ*^13^C and *δ*^15^N values compared to the body wall (respectively −14.4 ± 0.7‰ and 12.7 ± 0.6‰) while the feed had intermediate *δ*^13^C (−13.7 ± 0.05‰) and lower *δ*^15^N values (11.9 ± 0.03‰).

## 4. Discussion

Two out of the three co-culture conditions at sea showed the great potential of *H. forskali* to diversify shellfish aquaculture in the Northeast Atlantic, with an excellent survival rate (>90%) after one season of growth and promising specific growth rates. The association with PO condition was less efficient, but growth was still significant, and survival was excellent, which is encouraging. Juveniles reared in all three co-culture conditions clearly fed on contrasting food resources, revealing the adaptive trophic ability of *H. forskali*.

### 4.1. Rearing Conditions and Growth

Depth and hydrological conditions strongly differed between the three co-culture conditions at sea. With EA condition and underneath European oysters (EO condition), sea cucumbers were ranched at 8–12 m deep in relatively quiet waters (nearly no current and greatly reduced wave influence), while underneath PO, they were stocked in the near infralittoral zone, which remains always immersed but strongly subjected to the movements of waves especially in this mega tidal environment (up to 14 m tidal variations during spring tides). Stronger currents and the constant swaying of waves may have deprived them of feeding opportunities as they are constantly dislodged off of grazing on periphyton on the cage walls, as previously observed for juveniles of *Holothuria scabra* in floating hapa [29]. The species might also not be comfortable with such conditions since *H. forskali* in the wild is found on a rocky or granular substrate providing shelter to currents and waves and does not tolerate emersion. Higher densities in PO may also have contributed to lower feeding opportunities compared to other systems.

Our experiments were opportunistically conducted in conditions requiring low extra workload for producers, and thus, the SGR we obtained could probably be optimised. Yet, growth rates in EA (0.88% d^−1^) and EO conditions (0.94% d^−1^) were already within the range of SGR for juveniles *Apostichopus japonicus* (0.6%–2.7% d^−1^ for initial weights of 0.02–36 g; reviewed by Yokoyama [30]), whose aquaculture is the best mastered among temperate sea cucumbers. These rates are also similar to SGR obtained in recirculating aquaculture systems with juveniles *H. arguinensis* of comparable size (0.3%–1.1% d^−1^ for 2–8 g juveniles over a 4 months experiment) [31]. The SGR obtained in IM (2.76% d^−1^) was expected to be higher than that of the co-cultured *H. forskali*, as the initial weight of individuals was smaller and the water temperature was higher (11−20°C at sea between May and September, https://marc.ifremer.fr). Yet, it reached the highest values reported by Yokoyama [30], suggesting interesting prospects for this species. We believe that these encouraging results are mostly due to the use of juveniles, while, to our observations, adults always tend to decline when placed in captivity. Yet, co-culturing sea cucumbers with shellfish rather than finfish also provides pseudofaeces to sea cucumbers [13]. These excreta may provide interesting nutrients that are easily available and/or support microbial communities, especially nutritive for sea cucumbers.

Finally, the low SGR in EA conditions during winter suggests that there might be seasonal variations in the growth dynamics of *H. forskali*. A decrease in growth rates may be related to a slower metabolism due to low water temperature (5−14°C at sea between October and April; https://marc.ifremer.fr), which will need further investigation in controlled conditions and/or to the availability of food during the cold season. It was recently demonstrated that mature *H. forskali* replenish their gonads quickly after spawning, possibly due to higher availability of food linked to the spring bloom [32]. This strategy implies that to achieve a successful reproduction in early spring, it is more efficient to develop gonadal material during a period of high food availability and carry it all year long than to count on the available nutrients during winter. This advocates for low food resources in winter and can explain part of the low juveniles' growth in winter.

### 4.2. Food Sources

Pigments, FAs, and stable isotopes all clearly show the different rearing groups of *H. forskali* juveniles fed on different food sources. The IM experiment confirms that (1) pigment profiles of the stomach contents resemble that of ingested food sources, with the notable presence of green algae biomarkers (chlorophyll *b*, lutein, and zeaxanthin) [22, 33] in samples of the IM condition; (2) FA signature of the body wall resembles that of ingested food sources, with the especially high abundance of bacterial (18 : 1*ω*7) and green algae (18 : 3*ω*3) biomarkers [34] compared to other experiments in samples of the IM condition; (3) isotopic values of the body wall are slightly above those of ingested food sources, with a trophic enrichment factor ≈0.4‰ for *δ*^13^C and ≈2‰ for *δ*^15^N, corresponding to the average values for all animals reported by McCutchan et al. [35].

No external food was added to the rearing conditions underneath oysters, while cages of the EA condition were regularly supplied with brown and red macroalgae required for the growth of EA. High levels of degraded brown algae biomarkers (fucoxanthin derivatives) in stomach contents of juveniles from the EA condition compared to EO and PO conditions (Figure 3) confirm that *H. forskali* ingested organic matter originating from the supplied brown macroalgae. Yet, pigments were less abundant in the EA condition than in the IM condition (Table 2), suggesting that macroalgae only partly contributed to the ingested food of *H. forskali* in the EA condition. In addition, no FA biomarkers can clearly distinguish the body wall of juveniles in experiment EA compared to those in EO and PO conditions and individuals from the wild (Figure 6). These results contrast to those in IM, where juveniles fed decomposed green macroalgae were clearly noticeable and suggest that although *H. forskali* in EA may ingest macroalgae, they do not dominantly contribute to their growth.

Juveniles from the two experiments with oysters (EO and PO conditions) ingested food sources rich in microalgae biomarkers (diatoxanthin and diadinoxanthin) [36] and grew on nearly similar carbon and nitrogen sources as indicated by their close *δ*^13^C and *δ*^15^N signatures, which markedly differed from the higher *δ*^13^C and lower *δ*^15^N values of juveniles from experiment EA (Figure 7). However, juveniles in EO and PO conditions are strongly diverged by their growth rates and by the FA composition of their body wall. The low total concentration of FA (Figure 4) and high relative abundance of the FA 20 : 4*ω*6 and 20 : 1*ω*9 (Table 4) confirm that juveniles of experiment PO were not feeding correctly. Triacylglycerol oxidation is actually expected to change the ratio of triacylglycerol to phospholipids during starvation. The FA 20 : 4*ω*6 is a common component of membrane phospholipids, and LC-MUFA are especially retained in animal tissues during starvation as a consequence of their solubility properties [37]. Increased relative proportions of FA 20 : 4*ω*6 and LC-MUFA may thus serve as indicators of undernourishment, as previously highlighted during the experimental fasting of a tropical gastropod [38].

Finally, although our results indicate that *H. forskali* juveniles ingest algae, they do not seem to rely on such food sources for their growth in co-culture conditions at sea. Juveniles are nearly never found in the wild and thus, their usual food sources are still unknown. Yet, these results are in line with a previous study on wild populations where adult *H. forskali* were found to ingest diatoms but not to digest them during gut transit [22]. They also follow the observations of Clifford et al. [39] and Manship [40], who detected no cellulase activity in the digestive tract of *H. forskali*, confirming that this species is not adapted to feed on fresh algae. Our results thus show that sea cucumbers do not compete with the different shellfish studied for food access, which is an essential criterion for their integration into IMTA. Instead, sea cucumbers may have benefitted from the organic matter from shellfish faeces and pseudofaeces and/or grazed the biofilm growing on the cage walls.

### 4.3. Nutritional Quality

Sea cucumbers are considered products of high nutritional quality due to their elevated content of proteins, low proportion of fat, and wide array of bioactive molecules [41]. High levels of glycine, glutamic acid, and aspartic acid are commonly measured in sea cucumbers [42, 43, 44, 45, 46]. Proportions of alanine and proline in *H. forskali* are within the range reported in tropical species [46] and in other European species of the genus *Holothuria* [42], which are slightly above the values generally reported in the temperate sea cucumber *A. japonicus* [44, 45]. Yet, the content of essential amino acids (EAA) was lower in *H. forskali* than in other temperate species, which generally ranges between 25% and 60% [42, 45, 46].

Higher total AA content and higher proportions of glycine and alanine in wild *H. forskali* compared to indiscernible groups of reared juveniles suggest that there are ontogenic changes in the protein assemblages constituting the sea cucumbers' body wall and/or that juveniles feed was deficient in both AA. Glycine being a major constituent of collagen, itself being mainly responsible for the unique texture of the sea cucumbers body wall [47], those changes in AA profile between wild-collected individuals and reared juveniles may have an influence on the attractiveness of the product to consumers. The most attractive processed sea cucumbers and optimal size for commercialisation will have to be determined in the future.

### 4.4. Prospects

In the IM experiment, juveniles most probably grew on the microbial community that developed on decaying macroalgae and/or associated meiofauna rather than on the macroalgae themselves, as previously observed for juveniles of *H. scabra* in sea pens enriched with dried *Sargassum* spp. [48]. Feeding trials comparing growth rates with fresh and decomposed green macroalgae also supported this hypothesis, with much higher growth rates when fed decomposed green macroalgae (Grys and Raymond, unpublished data). Understanding which compounds and/or microbial assemblages benefit to *H. forskali* in controlled experimental conditions may allow to a better partition of the contribution of shellfish faeces/pseudofaeces vs. cage walls biofilm on juveniles growth. Such partitioning will have to be solved in the future as one of the most important characteristics of an IMTA is that an aquatic species benefits from the waste of another species. Otherwise, the system is defined as a co-culture. This has important consequences since an IMTA system helps reducing waste of the principal production and therefore allows bioremediation, while co-culturing species further increases waste deposition on a specific site. Yet, if *H. forskali* grows on the biofouling colonising cage walls, this may benefit oysters production by reducing mesh clogging and anoxic conditions within the cages.

Our trials were conducted with the idea that juveniles have to be enclosed to facilitate the immersion and recovering of individuals and avoid escapees. Yet, the species *H. forskali* is known to live predominantly on rocky surfaces, in contrast to other European sea cucumbers, and also a candidate for aquaculture that generally lives on sandy and/or muddy surfaces, especially *H. tubulosa*, *H. poli*, *H. mammata*, and *H. arguinensis*. In our experiments, the seabed under shellfish cultures was mostly sandy/muddy without rocks and hard surfaces, which is not expected to be a suitable place to rear *H. forskali*. The cage walls may have constituted the hard substrates, allowing the bonding of sea cucumbers. Another approach could be to create hard substrate areas below the hanging cultures of shellfish and/or fish cages. The simple presence of nearby inadequate sandy/muddy substrate may suffice to cage the sea cucumbers on created hard substrates while the risk of predation is poor on holothurians [49]. Handling of sea cucumbers would be minor as the hard substrates would be stocked with juveniles and only harvested once they have reached commercial size. This approach was indeed examined by Kim et al. [9] in polyculture systems involving hanging cultures of oysters (*Crassostrea gigas*) or sea squirts (*Halocynthia roretzi*) and the sea cucumber *A. japonicus*, with promising results. To date, bivalve production in France is mostly concentrated on the foreshore, and its extension is limited due to usage conflicts [6, 50]. Moving shellfish production to the subtidal offshore environment is considered to be a possible solution to this problem [51], and diversification prospects, along with the use of the entire water column for production, bring new arguments to support this expansion.

## 5. Conclusion

The first results for the growth of sea cucumber juveniles in the Northeast Atlantic are promising, but further research will still have to confirm that *H. forskali* can reach commercial size in these experimental systems. In addition, although the species is not new for the European market [42, 43], commercialisation pathways in France are still to be developed. Valuation opportunities and workload for producers will determine the future of sea cucumber aquaculture in multi-trophic systems of the Northeast Atlantic.

## Figures and Tables

**Table 1:** Synthesis of culture systems and growing performances of *H. forskali* juveniles cultured at sea and in indoor monoculture.

Co-culture species	Co-culture method	Culture containers	Water depth	Water temperature (°C)	Beginning of the trial	End of the trial	Trial duration (months)	Initial *(n)*	Final (*n*)	Survival (%)	Initial weight (g)	Initial density (kg m^−2^ of cage walls)	Initial density (kg m^−3^ of cage volume)	Final weight (g)	Growth rate (% d^−1^)
European abalone	Directly together	Two 1 m^3^ cubic polyethylene cages with 5 mm wide vents	8–12 m	5−14	25/11/2020	7/04/2021	4	80	74	93	6.2 ± 1.0	0.04	0.25	9.1 ± 2.8	0.28
				11−20	7/04/2021	2/11/2021	5				9.1 ± 2.8	0.06	0.36	33.2 ± 7.1	0.88
European oyster	Below oysters	Three 85 × 50 × 10 cm^3^ and 6 mm mesh size rigid plastic baskets	8–12 m	10−20	22/03/2021	16/09/2021	6	18	18	100	6.7 ± 1.2	0.04	0.95	35.3 ± 6.6	0.94
Pacific oyster	Below oysters	Nine 6 mm rigid mesh and 15 L Australian oyster baskets	Foreshore	12−20	29/04/2021	9/09/2021	4	81	80	99	7.5 ± 1.3	0.14	4.50	10.3 ± 3.1	0.24
None	Monoculture	Three 2.5 m long, 0.5 m wide raceway tanks	0.5 m	20	4/05/2021	6/09/2021	4	240	202	84	0.33 ± 0.04	0.04	0.04	10.3 ± 6.3	2.76

*n* = number of individuals.

**Table 2:** Pigment concentrations in stomach contents of *H. forskali* juveniles co-cultured at sea or in indoor monoculture and its feed.

Pigments (*μ*g g dw^−1^)	Co-cultured with European abalone (EA) (*n* = 7)	Co-cultured underneath European oysters (EO) (*n* = 3)	Co-cultured underneath Pacific oysters (PO) (*n* = 7)	Indoor monoculture (IM) (*n* = 8)	Indoor feed (IMF) (*n* = 2)
*β*-Carotene	732 ± 422	200 ± 78	57 ± 18	1,323 ± 428	1,551 ± 155
Canthaxanthin	89 ± 102	35 ± 20	74 ± 36	89 ± 38	14 ± 0
Chlorophyll *a*	15 ± 11	12 ± 15	8 ± 7	132 ± 67	1,035 ± 84
Chlorophyll *b*	3 ± 3	1 ± 0	0 ± 0	95 ± 42	266 ± 18
Diadinoxanthin	23 ± 10	229 ± 186	67 ± 38	5 ± 5	35 ± 2
Diatoxanthin	26 ± 8	264 ± 196	79 ± 57	42 ± 19	73 ± 3
Fucoxanthin	131 ± 98	70 ± 61	28 ± 18	101 ± 44	1,041 ± 111
Fucoxanthin derivatives	2,017 ± 750	258 ± 92	386 ± 203	168 ± 80	271 ± 23
Lutein	218 ± 204	20 ± 7	76 ± 58	7,454 ± 3,142	2,427 ± 176
Zeaxanthin	256 ± 60	131 ± 83	125 ± 112	5,470 ± 2,278	1,385 ± 218
Pheophorbide *a*	2,543 ± 1,844	703 ± 461	344 ± 92	29,698 ± 11,694	2,102 ± 152
Pheophytin *a*	19,344 ± 11,180	922 ± 459	539 ± 196	23,582 ± 7,391	8,993 ± 2,179
Total	25.4 ± 12.9	2.8 ± 0.4	1.8 ± 0.5	68.2 ± 24.6	19.2 ± 3.1

*n* = number of individuals analysed.

**Table 3:** Amino acids relative proportions (%) in the body wall of *H. forskali* juveniles co-cultured at sea, in indoor monoculture, and in adults gathered in the wild.

Amino acids (%)	Co-cultured with European abalone (EA) (*n* = 5)	Co-cultured underneath European oysters (EO) (*n* = 5)	Co-cultured underneath Pacific oysters (PO) (*n* = 5)	Indoor monoculture (IM) (*n* = 5)	Wild specimen (*n* = 5)
Alanine (Ala)	12.2 ± 0.7	12.0 ± 0.5	12.5 ± 0.7	11.7 ± 1.2	16.7 ± 1.4
Arginine (Arg)	2.1 ± 0.4	2.2 ± 0.3	1.9 ± 0.3	1.9 ± 0.5	2.1 ± 0.6
Aspartic acid (Asx)	9.8 ± 0.7	9.5 ± 0.2	9.7 ± 0.6	9.8 ± 0.6	7.4 ± 0.4
Glutamic acid (Glx)	14.8 ± 0.5	15.5 ± 0.7	14.5 ± 1.4	14.3 ± 0.7	11.9 ± 0.9
Glycine (Gly)	29.3 ± 1.6	29.8 ± 2.5	30.7 ± 2.3	29.2 ± 2.4	37.8 ± 4.1
Histidine (His)	0.4 ± 0.2	0.5 ± 0.2	0.5 ± 0.1	0.5 ± 0.1	0.2 ± 0.1
Isoleucine (Ile)	1.7 ± 0.1	1.6 ± 0.2	1.8 ± 0.4	1.7 ± 0.1	0.8 ± 0.3
Leucine (Leu)	2.8 ± 0.2	2.7 ± 0.3	2.6 ± 0.4	2.8 ± 0.2	1.6 ± 0.3
Lysine (Lys)	1.5 ± 0.1	1.5 ± 0.2	1.3 ± 0.3	1.5 ± 0.2	0.6 ± 0.2
Methionine (Met)	0.8 ± 0.1	0.9 ± 0.1	1.0 ± 0.3	0.8 ± 0.1	0.4 ± 0.1
Phenylalanine (Phe)	1.4 ± 0.1	1.1 ± 0.3	1.3 ± 0.3	1.4 ± 0.3	0.6 ± 0.2
Proline (Pro)	11.0 ± 1.8	10.2 ± 1.8	10.5 ± 1.2	11.6 ± 1.6	10.3 ± 2.9
Serine (Ser)	3.8 ± 0.4	3.8 ± 0.4	3.4 ± 0.8	3.8 ± 0.5	3.3 ± 0.4
Threonine (Thr)	4.6 ± 0.4	4.7 ± 0.4	4.3 ± 0.4	4.8 ± 0.2	3.7 ± 0.4
Tyrosine (Tyr)	1.1 ± 0.4	1.0 ± 0.1	1.3 ± 0.3	1.2 ± 0.3	0.5 ± 0.1
Valine (Val)	2.9 ± 0.3	2.9 ± 0.3	3.0 ± 0.5	2.9 ± 0.2	1.9 ± 0.6
Total EAA proportion (%)	16.1 ± 0.9	16.0 ± 1.1	15.6 ± 1.9	16.4 ± 1.0	9.9 ± 1.4
Total AA concentration (mg g^−1^)	249.4 ± 70.5	247.9 ± 42.0	165.0 ± 8.4	245.7 ± 73.0	465.0 ± 37.3

*n* = number of individuals analysed. EAA; essential amino acids.

**Table 4:** Fatty acid relative proportions (%) in the body wall of *H. forskali* in co-culture experiments, in indoor monoculture and in adults gathered in the wild.

Fatty acids (%)	Co-cultured with European abalone (EA) (*n* = 7)	Co-cultured underneath European oysters (EO) (*n* = 6)	Co-cultured underneath Pacific oysters (PO) (*n* = 7)	Indoor monocultured (IM) (*n* = 8)	Indoor monoculture feed (*n* = 2)	Wild specimen (*n* = 6)
Saturated						
14 : 0	3.4 ± 0.6	3.6 ± 0.6	1.4 ± 0.5	1.4 ± 0.3	8.1 ± 0.4	2.3 ± 0.9
15 : 0	0.9 ± 0.1	1.7 ± 0.2	0.8 ± 0.3	1.6 ± 0.4	1.9 ± 0.1	0.6 ± 0.2
16 : 0	6.9 ± 0.8	8.0 ± 0.6	5.1 ± 1.1	4.7 ± 0.9	30.1 ± 1.2	6.9 ± 1.6
18 : 0	5.3 ± 0.6	6.1 ± 1.0	4.6 ± 0.7	4.5 ± 0.6	3.2 ± 1.3	5.8 ± 0.8
19 : 0	1.1 ± 0.1	1.5 ± 0.1	1.3 ± 0.1	1.5 ± 0.1	0.1 ± 0.2	1.2 ± 0.1
20 : 0	3.2 ± 0.3	2.4 ± 0.1	2.4 ± 0.2	1.6 ± 0.1	0.5 ± 0.0	2.9 ± 0.4
21 : 0	1.2 ± 0.2	1.9 ± 0.3	2.1 ± 0.2	1.8 ± 0.2	—	1.6 ± 0.1
22 : 0	1.4 ± 0.1	1.3 ± 0.1	1.7 ± 0.2	1.4 ± 0.0	0.9 ± 0.2	1.3 ± 0.3
∑SFA	23.3 ± 1.9	26.5 ± 2.0	19.3 ± 2.3	18.5 ± 2.2	44.9 ± 2.5	22.5 ± 1.9
Monounsaturated						
16 : 1*ω*7	4.2 ± 0.2	6.1 ± 1.0	2.1 ± 0.7	2.8 ± 0.5	17.5 ± 0.4	3.3 ± 1.1
18 : 1*ω*9	4.4 ± 0.7	2.8 ± 0.4	2.9 ± 0.8	2.8 ± 0.5	2.7 ± 0.0	6.0 ± 3.0
18 : 1*ω*7	6.3 ± 0.3	5.5 ± 0.3	3.9 ± 0.5	7.5 ± 0.6	14.8 ± 0.5	3.9 ± 0.7
20 : 1*ω*9	9.8 ± 0.4	7.3 ± 0.6	11.0 ± 0.9	11.3 ± 0.8	0.2 ± 0.3	8.4 ± 1.1
20 : 1*ω*7	0.8 ± 0.0	1.0 ± 0.0	0.6 ± 0.1	0.6 ± 0.0	0.1 ± 0.2	0.6 ± 0.1
22 : 1*ω*11	0.4 ± 0.0	0.3 ± 0.0	0.4 ± 0.1	0.5 ± 0.1	—	0.6 ± 0.1
22 : 1*ω*9	2.1 ± 0.2	2.4 ± 0.4	2.8 ± 0.2	3.4 ± 0.5	—	1.4 ± 0.1
23 : 1*ω*9	4.4 ± 0.9	3.2 ± 0.3	6.3 ± 0.9	5.5 ± 0.6	—	6.5 ± 0.7
24 : 1*ω*9	3.4 ± 0.5	1.9 ± 0.4	2.5 ± 0.4	2.5 ± 0.2	—	2.2 ± 0.6
∑LC-MUFA	20.8 ± 1.2	16.1 ± 1.5	23.5 ± 2.3	23.9 ± 1.5	0.3 ± 0.5	19.6 ± 2.3
∑MUFA	35.6 ± 1.1	30.5 ± 0.5	32.4 ± 1.9	37.0 ± 1.7	35.5 ± 1.4	32.8 ± 2.3
Polyunsaturated						
18 : 3*ω*3	1.0 ± 0.1	0.7 ± 0.1	0.7 ± 0.2	3.8 ± 0.8	2.6 ± 0.2	0.5 ± 0.1
18 : 4*ω*3	0.9 ± 0.1	1.2 ± 0.4	0.4 ± 0.2	1.0 ± 0.2	0.9 ± 0.1	0.8 ± 0.2
20 : 5*ω*3	11.4 ± 0.5	14.9 ± 0.9	14.7 ± 1.0	14.3 ± 2.0	1.7 ± 0.2	12.7 ± 1.6
22 : 6*ω*3	1.3 ± 0.1	4.0 ± 0.5	1.9 ± 0.6	0.9 ± 0.1	0.2 ± 0.3	1.9 ± 0.5
∑*ω*3	14.6 ± 0.6	20.8 ± 1.3	17.7 ± 1.6	19.9 ± 2.4	5.4 ± 0.8	15.9 ± 1.3
18 : 2*ω*6	1.5 ± 0.2	1.1 ± 0.1	1.2 ± 0.3	1.8 ± 0.3	1.7 ± 0.1	1.7 ± 0.4
20 : 2*ω*6	1.4 ± 0.1	1.2 ± 0.2	1.7 ± 0.1	2.0 ± 0.2	—	1.5 ± 0.1
20 : 4*ω*6	18.7 ± 2.2	13.7 ± 2.5	23.8 ± 2.4	13.8 ± 2.4	0.8 ± 0.1	21.1 ± 2.4
22 : 5*ω*6	0.6 ± 0.1	1.1 ± 0.1	1.1 ± 0.1	0.5 ± 0.1	—	1.3 ± 0.4
∑*ω*6	22.2 ± 2.2	17.1 ± 2.7	27.9 ± 2.6	18.2 ± 2.2	2.5 ± 0.2	25.7 ± 2.7
∑HUFA	32.1 ± 2.2	33.7 ± 2.4	41.5 ± 1.8	29.4 ± 2.6	2.7 ± 0.7	37.0 ± 3.8
∑PUFA	36.9 ± 2.0	37.9 ± 2.2	45.5 ± 1.6	38.1 ± 2.1	8.0 ± 1.0	41.5 ± 3.6
Branched						
15 : 0iso	2.3 ± 0.5	3.0 ± 0.5	1.5 ± 0.5	4.3 ± 0.6	8.1 ± 0.2	1.6 ± 0.8
15 : 0anteiso	0.9 ± 0.3	0.8 ± 0.1	0.7 ± 0.2	1.3 ± 0.2	2.7 ± 0.0	0.7 ± 0.4
16 : 0iso	1.0 ± 0.2	1.3 ± 0.2	0.5 ± 0.2	0.8 ± 0.1	0.9 ± 0.0	0.9 ± 0.4
∑BrFA	4.2 ± 1.0	5.1 ± 0.9	2.7 ± 0.9	6.4 ± 0.9	11.7 ± 0.2	3.1 ± 1.6
Total FA concentration (mg g^−1^)	11.8 ± 1.5	10.7 ± 2.7	7.8 ± 2.6	9.5 ± 1.2	13.0 ± 2.5	4.6 ± 1.0

—; not detected, SFA; saturated fatty acids, MUFA; monounsaturated FA, LC-MUFA; long-chain MUFA (≥20 carbons chain length), PUFA; polyunsaturated FA, HUFA; highly unsaturated FA (≥20 carbons chain length and three double bounds), BrFA; branched FA.

## Data Availability

The datasets and R script generated and analysed during the current study are available in the GitHub repository, https://github.com/DAVID-Fk/Sea-cucumber-juveniles.
